# Molecular Diagnosis, Prevalence and Importance of Zoonotic Vector-Borne Pathogens in Cuban Shelter Dogs—A Preliminary Study

**DOI:** 10.3390/pathogens9110901

**Published:** 2020-10-28

**Authors:** Adrian Alberto Díaz-Sánchez, Belkis Corona-González, Marina L. Meli, Lisset Roblejo-Arias, Osvaldo Fonseca-Rodríguez, Anisleidy Pérez Castillo, Ernesto Vega Cañizares, Evelyn Lobo Rivero, Regina Hofmann-Lehmann

**Affiliations:** 1 Centro Nacional de Sanidad Agropecuaria (CENSA), Carretera de Tapaste y Autopista Nacional, Apartado Postal 10, San José de las Lajas 32700, Cuba; add196@usask.ca (A.A.D.-S.); bcorona@censa.edu.cu (B.C.-G.); lroblejo@censa.edu.cu (L.R.-A.); anisleidy@inhem.sld.cu (A.P.C.); evega@censa.edu.cu (E.V.C.); elobo@censa.edu.cu (E.L.R.); 2 Clinical Laboratory, Department of Clinical Diagnostics and Services, and Center for Clinical Studies, Vetsuisse Faculty, University of Zurich, 8057 Zurich, Switzerland; mmeli@vetclinics.uzh.ch; 3 Department of Biology, University of Saskatchewan, 112 Science Place, Saskatoon, SK S7N 5E2, Canada; 4 Department of Epidemiology and Global Health, Umeå University, 901 87 Umeå, Sweden; osvaldo.fonseca@umu.se; 5 Instituto Nacional de Higiene, Epidemiología y Microbiología (INHEM), La Habana 10300, Cuba

**Keywords:** canine vector-borne diseases (CVBDs), *Anaplasma platys*, *Ehrlichia canis*, *Rickettsia felis*, *16S* rRNA, shelter dogs, real-time qPCR

## Abstract

The present study aimed to determine the prevalence of zoonotic vector-borne pathogens, including *Anaplasma platys*, *Anaplasma phagocytophilum*, *Borrelia burgdorferi* sensu lato, *Ehrlichia canis* and *Rickettsia* spp. in shelter dogs from Cuba. Blood samples were collected from 100 shelter dogs and examined by molecular methods. Overall, 85 (85%; 95% CI: 77.88–92.12) dogs tested positive for at least one vector-borne pathogen using species-specific qPCR assays. Among the positive samples, *E. canis* was the most prevalent 62% (95% CI: 52.32–71.68), followed by *A. platys* 40% (95% CI: 30.23–49.77) and *Rickettsia felis* 27% (95% CI: 18.15–35.85), whereas 36% (95% CI: 26.43–45.57) showed co-infections. All samples were negative for *A. phagocytophilum* and *B. burgdorferi* s.l. The presence of 248 *Rhipicephalus sanguineus* ticks collected from the dogs was not statistically associated with the occurrence of infections. Thrombocytopenia was the most frequent haematological alteration found in PCR-positive dogs; it was statistically associated with the presence of *E. canis*, as well as co-infections (*p* < 0.05). The phylogenetic analyses of *A. platys* and *E. canis* based on *16S* rRNA, *groEL* and *gltA* genes showed a low genetic diversity between Cuban strains. The present study demonstrates the high prevalence of vector-borne pathogens with zoonotic potential in shelter dogs from Cuba.

## 1. Introduction

Canine vector-borne diseases (CVBDs) consist of a group of infectious diseases caused by a range of pathogens transmitted by arthropod vectors, including ticks, mosquitoes, fleas and lice [1]. Clinical signs commonly associated with these diseases include anorexia, pyrexia, lethargy, weight loss, bleeding disorders and icterus progressing to fatal outcomes in some dogs [2]. In addition, some CVBD-causing pathogens are a cause of major zoonotic concern and constitute a serious human health hazard worldwide [1].

The Anaplasmataceae family are vector-transmitted bacteria that infect a variety of vertebrate hosts, including the tick-borne pathogens *Ehrlichia canis* and *Anaplasma platys*, which are obligatory intracellular bacteria of monocytes and platelets, respectively [3]. *Ehrlichia canis* infection has a worldwide distribution and is the agent of canine monocytic ehrlichiosis (CME) in dogs, wolves and jackals. Infections with *E. canis* have become a public health concern, since an organism genetically and morphologically similar to *E. canis* was suggested to infect humans in Venezuela [4] and Costa Rica [5]. *Anaplasma platys* infection, also described around the world, causes canine infectious cyclic thrombocytopenia (CCT) in dogs [6]. The pathogen has also been identified in a broad range of other hosts than dogs, including cats [7], cattle [8], foxes [9] and humans [10]. Single infections with *A. platys* are usually mild or asymptomatic, although may progress to severe or fatal in some cases, particularly when coinfections with other tick-borne pathogens such as *E. canis* are involved [6]. The brown dog tick *Rhipicephalus sanguineus* is the recognized vector of *E. canis* and the suspected vector of *A. platys* [11].

Lyme borreliosis (LB) is the most prevalent tick-borne zoonotic disease in the northern hemisphere (~130,000 human cases per year) and is caused by the Gram-negative bacteria of the *Borrelia burgdorferi* sensu lato (s.l.) complex in Europe, North America, and Asia [12]. Human granulocytic anaplasmosis (HGA) is another tick-borne disease with public health importance, which is caused by the obligate intracellular bacterium *Anaplasma phagocytophilum* [13]. Fatal outcomes have been observed in immunocompromised individuals [14]. Coexistence of *A. phagocytophilum* with *B. burgdorferi* s.l. is attributed to common vectors, *Ixodes ricinus* in Europe, *Ixodes scapularis* in North America, and *Ixodes persulcatus* in Asia [13]. Moreover, rickettsiosis is a disease caused by bacterial species belonging to the genus *Rickettsia* (order Rickettsiales, family Rickettsiaceae), which are widely distributed throughout the world, and several of these species are well-known emerging or re-emerging zoonotic pathogens transmitted by bloodsucking arthropods, mainly ticks, but also fleas, mites and lice [15]. Typically, clinical symptoms associated with rickettsioses are not specific and can lead to serious complications when misdiagnosed resulting in marked morbidity, including acute renal failure, meningoencephalitis, gastrointestinal bleeding, and multiple organ failure with occasional fatalities [16].

The diagnosis of CVBDs represent a substantial challenge for veterinarians due to similar and mainly unspecific clinical signs induced by several vector-borne pathogens; further, co-infections with two or more pathogens may influence clinical signs and laboratory changes, thereby complicating the diagnosis [17]. Different techniques including indirect (serology) or direct (e.g., blood smears and PCR) methods are used as diagnostic tools for CVBDs. Serologic tests such as IFAT, ELISA, and commercial dot-ELISA tests (Snap3D×, Snap4D×) are commonly used for diagnosis [18]. However, serology usually shows cross-reactivity between antigenically closely related pathogens, and this method does not differentiate between current infection and previous exposure to agents [19]. Direct detection methods, such as blood smear examination often shows limited sensitivity and poor specificity as it cannot reliably identify the species, besides this, the finding of intracellular inclusions is difficult and time consuming [19]. Conversely, a molecular approach, i.e., PCR, is a more sensitive and specific assay than the others due to its ability to distinguish between closely related pathogens species and to reveal the current infections [8]. Positive PCR results confirm infection, and further molecular characterization allows for the comparison of strains from different regions of the world [20]. 

The presence of canine tick-borne pathogens *A. platys* and *E. canis* have been previously described in Cuba [21,22], but the information regarding the prevalence and genetic diversity of these pathogens remains lacking. The present study aimed to determine the prevalence of *A. platys*, *A. phagocytophilum*, *B. burgdorferi* s.l., *E. canis* and *Rickettsia* spp. infections in Cuban shelter dogs by means of probe-based TaqMan^®^ real-time qPCR assays and DNA sequencing analysis, and to evaluate the occurrence of haematological disorders in infected dogs.

## 2. Results

**Figure 1:**
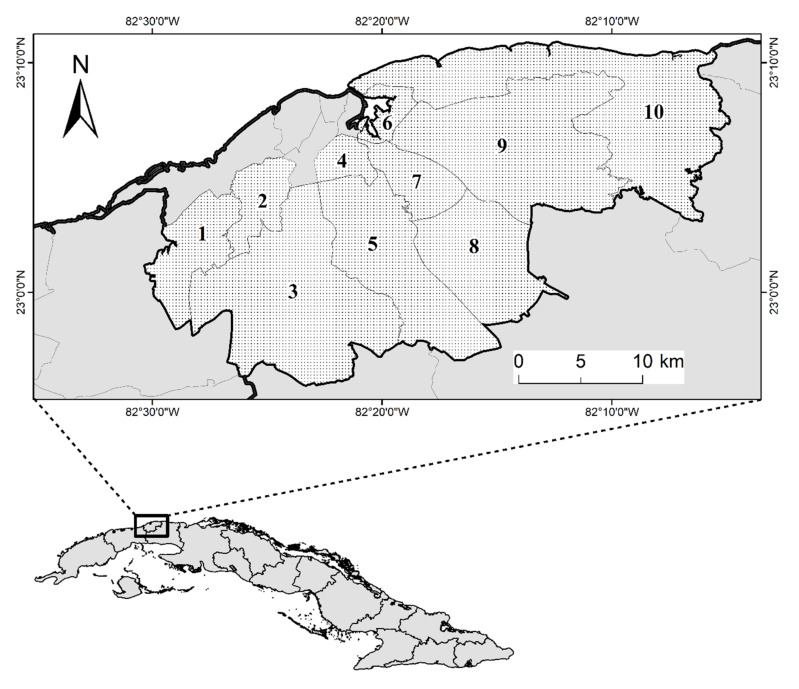
Map of study area. Location of the municipalities whereby the sample collections were conducted in the province of Havana City, Cuba, which included 1. La Lisa; 2. Marianao; 3. Boyeros; 4. Diez de Octubre; 5. Arroyo Naranjo; 6. Regla; San Miguel del Padrón; 8. Cotorro; 9. Guanabacoa; and 10. Habana del Este. Scale bar = 10 km.

In total, 100 dog blood samples were collected from 11 municipalities in the Havana province (Figure 1). Using sensitive species-specific PCR assays and sequence confirmation, *E. canis*, *A. platys*, and *Rickettsia felis* were detected in dogs. Neither *A. phagocytophilum* nor *B. burgdorferi* s.l. DNA was identified in any of the dog blood samples included in this study. Out of 100 blood samples, 85 (85%; 95% CI: 77.88–92.12) tested positive for at least one vector-borne pathogen using real-time qPCR assays. Among these positive samples, *E. canis* was the most prevalent 62% (95% CI: 52.32–71.68), followed by *A. platys* 40% (95% CI: 30.23–49.77) and *Rickettsia* spp. 27% (95% CI: 18.15–35.85). Dogs were most often co-infected with *E. canis* and *A. platys* in 28 (28%; 95% CI: 19.05–36.95), followed by *E. canis* and *Rickettsia* spp. in 13 (13%; 95% CI: 6.29–19.71), *A. platys* and *Rickettsia* spp. in 11 (11%; 95% CI: 4.76–17.24), and triple mixed infections were detected in 8 (8%; 95% CI: 2.59–13.41) dogs. The results from the real-time qPCR testing are summarized in Table 1. 

Tick infestation was observed in 57 out of 100 sampled dogs, and a total of 248 ticks were submitted for identification to species level. All the ticks collected were identified morphologically as *R. sanguineus*, and consisted of 111 females, 131 males and 6 nymphs. The presence of ticks was not statistically associated with the occurrence of PCR-positives infections (*p* = 0.115), i.e., *E. canis* (*p* = 0.269), *A. platys* (*p* = 0.268), *Rickettsia* spp. (*p* = 0.814) and co-infections (*p* = 0.411) (Supplementary Table S1). Most of the collected ticks were visibly engorged with blood. Ticks were collected throughout the year, and adult ticks were seen in every month during the sample collection.

A complete blood count (CBC) was available for 90 of the 100 sampled dogs. Table 2 shows the main statistics values obtained from the haematological parameters determined for the tested animals, which were distributed into five groups, including *E. canis*-, *A. platys*-, *Rickettsia* spp.-PCR positive, co-infected and non-infected dogs. The most common haematological abnormalities among tested dogs included thrombocytopenia (54/90, 60%; 95% CI: 49.68–70.32), anaemia (43/90, 47.78%; 95% CI: 37.26–58.3), leukopenia (10/90, 11.11%; 95% CI: 4.49–17.73) and leucocytosis (9/90, 10%; 95% CI: 3.68–16.32) (Supplementary Table S2). Although thrombocytopenia (50/85, 58.82%; 95% CI: 48.15–69.5) and anaemia (38/85, 44.71%; 95% CI: 33.92–55.49) were more frequent in PCR-positive dogs, for red blood cell count (RBC), haemoglobin concentration (Hb), haematocrit (HCT), mean corpuscular volume (MCV), mean corpuscular haemoglobin (MCH), mean corpuscular haemoglobin concentration (MCHC), and total white blood cell counts (WBCs), there were no statistically significant differences between the five groups (*p* > 0.05). However, the mean values of platelet counts in *E. canis*-PCR positive (*p* = 0.018) and co-infected dogs (*p* = 0.016), as well as MCH (*p =* 0.042) and MCHC (*p* = 0.027) values for co-infected dogs were statistically different from non-infected animals (*p* < 0.05). 

The sequence analysis of the nearly full-length *16S* rRNA gene sequences obtained from *E. canis* (1434 bp) and *A. platys* (1431 bp) Cuban isolates revealed high identities >99% with several sequences of *E. canis* (e.g., LC269822, EF139459) and *A. platys* (e.g., EU106856, CP000107) available in GenBank, respectively. The nucleotide sequences obtained in the present study were not 100% identical to each other and may represent local variants that exist within the studied region. In addition, partial *E. canis*-*gltA* (507 bp), *A. platys*-*groEL* (625 bp) and *Rickettsia* spp.-*htrA* (434 bp) gene sequences were obtained from PCR-positive samples. The sequences obtained from *ompA* and *ompB* fragment genes were not evaluable. The *E. canis*-*gltA* obtained sequences were 100% identical to each other and to reported sequences from China (CP025749), the Philippines (LC428206) and Zambia (LC373038), while, for *A. platys*-*groEL*, sequences were 100% identical to each other and to reported sequences from the Democratic Republic of Congo (AF478129), Japan (AY077621) and Venezuela (AF399916). The *Rickettsia* spp.-*htrA* (434 pb) were 100% identical to each other and showed high identities >99% with the 17 kDa surface antigen gene sequences of *R. felis* type reference isolates reported from Mexico (GU447234) and the USA (CP000053). For *E. canis*-*gltA*, *A. platys*-*groEL* and *R. felis*-*htrA* gene sequences no nucleotide variation was observed among the sequenced PCR amplicons. 

**Figure 2:**
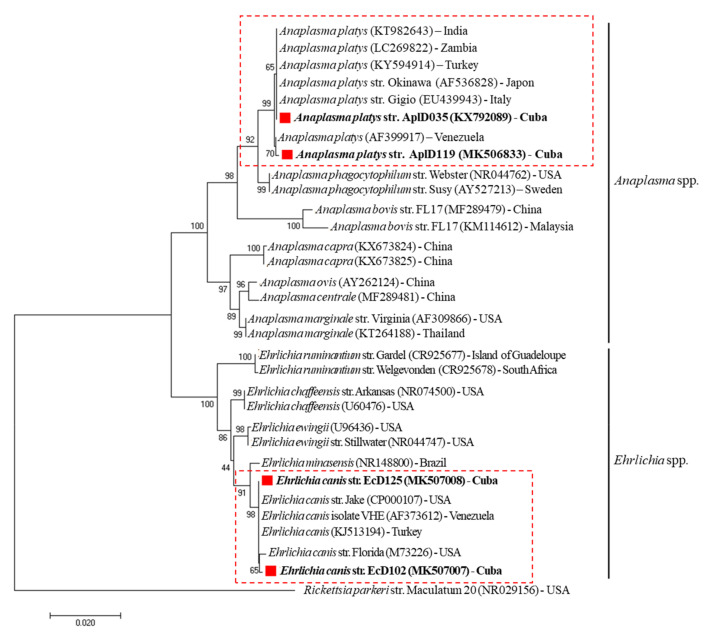
Phylogenetic analysis of *A. platys* and *E. canis* strains identified in shelter dogs from Cuba. The neighbor-joining (NJ) phylogenetic tree was constructed based on the Kimura 2-parameter model using the 16S rRNA gene sequences from *A. platys* and *E. canis* strains identified in Cuba and other members of the family Anaplasmataceae. The internal nodes indicate the percentages of 1000 bootstrap replicates that supported the branch. *Rickettsia parkeri* (NR029156) was used as an outgroup. GenBank accession numbers and country of origin are shown. The *A. platys* (KX792089, MK506833, MK506834) and *E. canis* (MK507007, MK507008, MK507009) 16S rRNA gene sequences obtained in this study are indicated with “red squares and bold text”.

**Figure 3:**
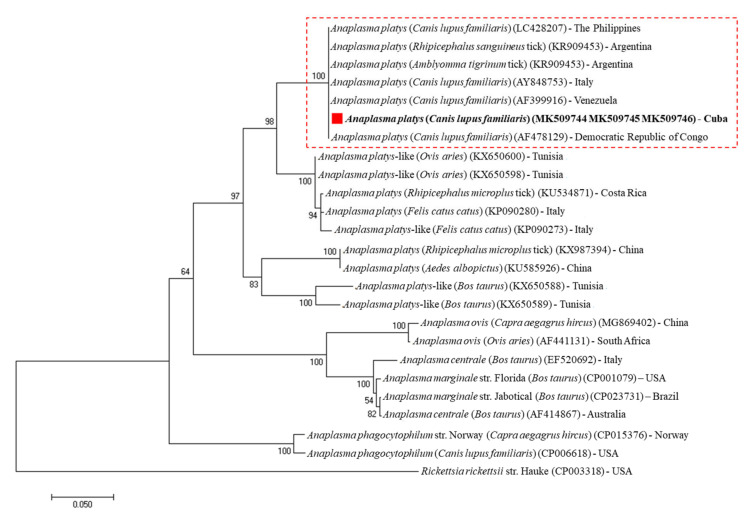
Phylogenetic analysis of *A. platys* strains identified in Cuba based in *groEL* gene sequences. The neighbor-joining (NJ) phylogenetic tree was constructed based on the Tamura 3-parameter model using the *groEL* gene sequences from *A. platys* strains identified in Cuba and other members of the genus *Anaplasma*. Posterior probability values are shown on the branches. *Rickettsia rickettsii* (CP003318) was used as an outgroup. GenBank accession numbers, host and country of origin are shown. The *A. platys* (MK509744, MK509745, MK509746) *groEL* gene sequences obtained in this study are indicated with “red squares and bold text”.

**Figure 4:**
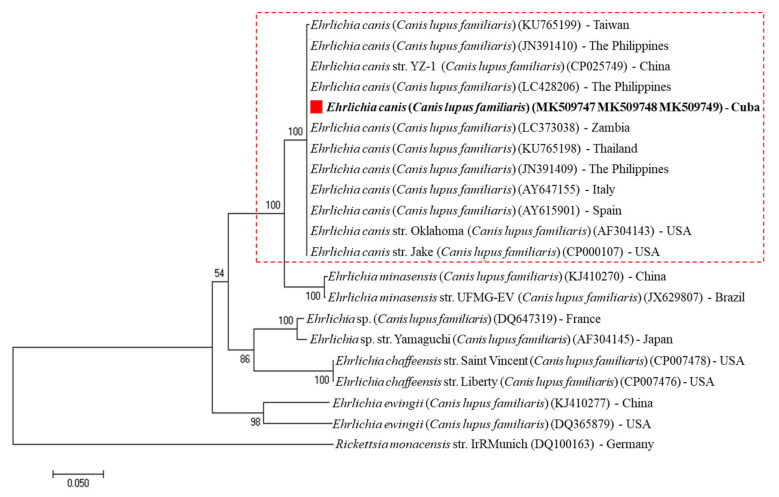
Phylogenetic analysis of *E. canis* strains identified in Cuba based in *gltA* gene sequences. The neighbor-joining (NJ) phylogenetic tree was constructed based on the Tamura 3-parameter model using the *gltA* gene sequences from *E. canis* strains identified in Cuba and other members of the genus *Ehrlichia*. Posterior probability values are shown on the branches. *Rickettsia monacensis* (DQ100163) was used as an outgroup. GenBank accession numbers and country of origin are shown. The *E. canis* (MK509747, MK509748, MK509749) *gltA* gene sequences obtained in this study are indicated with “red squares and bold text”.

The phylogenetic analysis based on *16S* rRNA gene sequences were grouped into two main clades of *Anaplasma* spp. and *Ehrlichia* spp. In addition to *A. platys* and *E. canis* strains, closely related species of the tick-borne parasites were included, such as *Anaplasma bovis*, *Anaplasma centrale*, *Anaplasma capra*, *Anaplasma marginale*, *Anaplasma ovis*, *Anaplasma phagocytophilum*, *Candidatus Anaplasma camelii, Ehrlichia chaffeensis*, *Ehrlichia ewingii*, *Ehrlichia muris*, *Ehrlichia minasensis* and *Ehrlichia ruminantium*. A biologically divergent member of the family *Anaplasmataceae*, *Rickettsia parkeri* was used as an outgroup. As expected, the resultant phylogenetic tree revealed that *A. platys* and *E. canis* Cuban isolates were clustered tightly with other *A. platys* and *E. canis* strains reported around the world, respectively (Figure 2). In addition, phylogenetic analysis based on the alignment of the *A. platys*-*groEL* partial gene sequences obtained in this study was compared with several *A. platys* reference sequences, and other *Anaplasma* spp. found in GenBank. *A. platys*-*groEL* Cuban genotype was tightly classified in *A. platys* cluster grouped with other strains isolated from different host species worldwide, supported with 100% bootstrap value (Figure 3). Moreover, phylogenetic tree based on the alignment of *gltA* partial gene sequences of *Ehrlichia* spp. found in GenBank shows the presence of five clusters represented by *E. canis*, *E. chaffeensis*, *E. ewingii*, *E. minasensis*, *Ehrlichia* sp. and *Rickettsia monacensis* as an outgroup. The *E. canis*-*gltA* Cuban strain was grouped within the *E. canis* clade formed by strains isolated from different host species worldwide, supported with 100% bootstrap value (Figure 4).

## 3. Discussion

To the authors’ knowledge, this is the first study addressed to investigate canine arthropod-borne pathogens in stray dogs housed in animal shelters from Cuba, and demonstrated that rescued dogs housed in shelters from the investigated areas showed high prevalence rates for several arthropod-borne pathogens. Eighty five percent of the dogs tested PCR-positive to pathogenic organisms. This finding can be easily explained since stray dogs usually are neither protected by preventive measures against ectoparasites nor receive any proper veterinary care, and therefore are at increased risk of infections by arthropod-borne pathogens. Importantly, all infections detected here have a relevant zoonotic potential since *E. canis*, *A. platys* and *Rickettsia* spp. human infections have been reported from Venezuela [4], Grenada [23], and Spain [24], respectively.

The sample size in the current study was rather small. Nonetheless, it is of importance to note that the overall prevalence infection rate with at least one zoonotic pathogen (i.e., *E. canis*, *A. platys* and *Rickettsia* spp.) recorded during this study was higher than that reported in previous molecular studies conducted in dogs from Italy (44/145; 30.3%) [25], Thailand (78/181; 43.1%) [26], Brazil (118/181; 65.2%) [27], and Haiti (111/207; 53.6%) [28]. This worldwide variation in infection rates are likely attributed to several factors, including the demography of dog populations, the type and number of samples analysed, the extent of tick infestations, and the sensitivities of diagnostic methods employed [29]. *E. canis* and *A. platys* were the most prevalent pathogens (62% and 40%, respectively), while *Rickettsia* spp. was less frequently detected (27%). Moreover, *E. canis* and *A. platys* were the most frequently detected either as single infections (29% and 9%, respectively) or as coinfections (28%). Coinfections with multiple tick-borne pathogens in dogs are quite frequently reported [30] and often occur due to concomitant transmission by the same tick vector, *R. sanguineus* [31], which was the only tick species found in this study. The presence of co-infections is clinically important because it may pose a diagnostic and therapeutic problem as infected dogs frequently show unspecific symptoms, such as fever, weight loss, inappetence, lethargy or apathy, which give no indication of the possible causative agent [2]. Although in this study a high prevalence of mixed infections was observed, the sampled dogs showed no clinical signs consistent with *E. canis*, *A. platys* and *Rickettsia* spp. infections more than pale mucosa, anorexia, apathy, dehydration and poor body condition. Most of the dogs were asymptomatic, which may reflect the existence of chronic, subclinical or mild infections that makes the clinical diagnosis of infected dogs in the studied region difficult [32]. 

Prior to this study, Navarrete et al. [21] described the presence of E. canis as a canine tick-borne pathogen in Cuba; however, to date, the thrombocytopenia in dogs was a problem of unknown aetiology. In this study, the presence of *E. canis* infections was associated with significantly lower platelet count values compared to non-infected dogs (*p* = 0.018), this fact was even more significant when co-infection was considered (*p* = 0.016). In general, this result is consistent with several studies carried out under both natural and experimental conditions, which concluded that thrombocytopenia is the major haematological abnormality associated with *E. canis* infections [33]. The presence of thrombocytopenia is commonly used alone as a useful haematological marker of *E. canis* infection for the diagnosis of CME. However, a previous study conducted by Santos et al. [34] demonstrated that the diagnosis of *E. canis* infection in dogs just based on the occurrence of thrombocytopenia is not sufficient, and screening for other tick-borne pathogens such as *A. platys* and *Babesia* spp. is recommended to reach a definite diagnosis.

The resultant phylogenetic analysis based on the nearly full length *16S* rDNA sequences revealed that *A. platys* and *E. canis* strains from Havana, Cuba, were tightly grouped with other *A. platys* and *E. canis* isolates from dogs around the world (Figure 2). These results are consistent with a previous report described by de la Fuente et al. [35], which support the hypothesis that *A. platys* strains are neither geographically nor host segregated. In addition, a highly conserved genetic profile was observed for the *A. platys* and *E. canis* strains based on the *groEL* and *gltA* partial gene sequences analysis, respectively. Sequence analysis of the *E. canis*-*gltA* and *A. platys*-*groEL* genes performed on three Cuban strains revealed 100% identity, even though the analysed samples were obtained from different areas. The sequence alignments and phylogenetic trees suggested little genetic diversity and homogeneous evolution within *A. platys* and *E. canis* strains, based on the close similarity amongst their *16S* rDNA, *groEL* and *gltA* sequences from geographically diverse areas studied in this report. The results obtained were in concordance with previous reports of slight genetic variation between sequenced genes from different *A. platys* and *E. canis* strains [36,37]. The choice of molecular markers with an appropriate mutation rate is an essential step in phylogenetic analysis. Consistent with previous investigations conducted by Marsilio et al. [38] and Ben Said et al. [20], nucleotide variability of both *groEL* and *gltA* genes have proved be useful as markers to clarify evolutionary relationship and correct identification among *Anaplasma* ssp. and *Ehrlichia* spp., respectively. These conclusions are consistent with other reports, in which *gltA* and *groEL* genes indicated higher interspecies nucleotide variability than that observed for the *16S* rRNA gene [38,39]. However, further studies are needed in Cuba to investigate the genetic variability among different *A. platys* and *E. canis* strains. 

The presence of *A. phagocytophilum* and *B. burgdorferi* s.l. DNA was not identified in any of the blood samples tested. The negative results for *A. phagocytophilum* and *B. burgdorferi* s.l. is in accordance with the absence of the main vector of these pathogens, which are hard ticks other than *R. sanguineus* that have never been reported in Cuba [40]. Regarding *B. burgdorferi* s.l., there is a previous report in Cuba by Rodríguez et al. [41] that described the detection of antiborrelial antibodies and clinical signs resembling Lyme disease in humans, but, according to our research, the existence of *B. burgdorferi* s.l. still remains unproven.

Twenty-seven (27%) of 100 samples were positive in the *Rickettsia* species DNA screening qPCR, and the subsequent sequence analysis identified *R. felis* presence. The qPCR assay used in the present study was developed by Stenos et al. [42], and can detect most Rickettsia species in the spotted fever and typhus groups with a high specificity and sensitivity, capable of detecting one target copy gene per PCR reaction. However, despite a high prevalence of *Rickettsia* species found in Cuban dogs by *gltA* gene real-time qPCR, we were only capable of obtaining a partial Rickettsia-*htrA* gene sequence by conventional PCR. Unfortunately, rickettsial DNA could not be amplified in any of the samples when tested by PCR based on *ompA* and *ompB* genes, limiting additional phylogenetic inferences. The variable successful amplification of different genes is likely explained by the fact that the molecular detection of rickettsial DNA from blood samples based on conventional PCR shows low sensitivity. This point of fact is due to the pathogenic mechanisms of *Rickettsia* spp. that once infect endothelial cells, the bacterial load in blood is decreased until too low numbers, which makes them incapable of being detected by molecular analysis [43]. This is the first detection of *R. felis*, a member of the spotted fever group Rickettsia (SFGR), infections in dogs from Cuba. A study conducted by Ng-Nguyen et al. [44] demonstrates based on molecular evidence the role of the domestic dog (Canis lupus familiaris) as a mammalian reservoir for R. felis and as a potential source of human rickettsial infection. A previous study conducted by Noda et al. [45] described the detection of “Candidatus Rickettsia amblyommii” in Amblyomma mixtum tick species by PCR, which constitutes the first report of an SFGR member in Cuba. The high prevalence of *R. felis* found in the tested Cuban dogs highlights the substantial importance of this pathogen on human health, since Rickettsiosis has become a re-emerging problem worldwide and suggests it may be causing unreported or unstudied SFGR in Cuba. The SFGR infection among dogs in Cuba was interesting given the findings that include descriptions of the occurrence and clinical significance of *R. sanguineus*-associated Rickettsia infections in dogs and humans [46]. These findings indicate the need for further studies regarding the presence of *Rickettsia* spp. in *R. sanguineus* from Cuba.

## 4. Materials and Methods 

### 4.1. Sample Collection and DNA Extraction 

The sample collection was performed between September 2016 and August 2017 in animal shelters housing dogs from ten municipalities of Havana City, Cuba (Figure 1). The climate of this region is tropical and humid with two marked climatic periods, a dry season from November to April with temperatures varying from 15 to 26 °C, and a wet season from May to October with temperatures typically range between 22 and 32 °C. The annual average temperature varies between 22 and 28 °C, and relative humidity of 80%. As is typical of most animal shelters in Cuba, the population included stray dogs and dogs abandoned by their owners for various reasons. Whole blood samples were collected from 100 randomly selected dogs of different breeds, sex and age. Samples were drawn from the jugular vein using sterile Vacutainer needles and K2EDTA-coated tubes (Becton-Dickinson Vacutainer Systems, Franklin Lakes, NJ, USA), and maintained at 4 °C until DNA extraction within 24 h of blood collection, which was performed using the Wizard Genomic DNA Purification Kit (Promega, Madison, WI, USA) according to the manufacturer’s instructions. The DNA samples were eluted in 100 µL of DNA Rehydration Solution and stored at −20 °C until used as template for polymerase chain reaction (PCR) assays. An extraction control (DNA-free distilled water) was included for every 20 samples extracted. Sampled dogs were subjected to a thorough external physical exam looking for the presence of ticks, including their ears, heads, necks, chests, bellies, and paws. A representative sample of up to ten ticks was manually removed per infested dog using forceps and ensuring that the mouth parts remained intact. All collected specimens were deposited in labelled plastic tubes, covered by a piece of cloth, secured by rubber band, and transported alive to the laboratory for identification using a stereomicroscope (Carl Zeiss AG, Oberkochen, Germany) according to the standard taxonomic key described by Estrada-Peña et al. [47]. Once identified, the ticks were preserved in 70% ethanol (Merck®, Kenilworth, NJ, USA) using 1.5 mL plastic sterile.

### 4.2. Haematological Parameters

Complete blood counts (CBCs) were performed on 90 out of the 100 EDTA-anticoagulated blood samples within 24 h of blood collection using an automated haematological cell counter ABX Micros ESV 60 (Horiba, Kyoto, Japan). The parameters evaluated in the hemogram included haematocrit (HCT), haemoglobin concentration (Hb), red blood cell count (RBC), mean corpuscular volume (MCV), mean corpuscular haemoglobin (MCH), mean corpuscular haemoglobin concentration (MCHC), total white blood cell counts (WBCs), and total platelet counts (PLTs). 

### 4.3. PCR Amplification and Sequencing

To verify the presence of amplifiable DNA in the samples, a real-time qPCR assay for the canine housekeeping gene glyceraldehyde-3-phosphate dehydrogenase (GAPDH) was performed as previously described by Sieber-Ruckstuhl et al. [48]. All DNA samples were analysed by real-time qPCR using the primers and probe set previously described for *A. phagocytophilum* [49], *A. platys* [50], *B. burgdorferi* s.l. [51], *E. canis* [52] and *Rickettsia* spp. [42]. The PCR reactions included 500 nM of each primer, 250 nM probe, 0.2 µL of Uracil-DNA Glycosylase (UNG, Eurogentec S.A., Seraing, Belgium), 10 µL of the qPCR Mastermix (Eurogentec S.A., Seraing, Belgium) and 5 μL of DNA in a final volume of 20 μL. All real-time qPCR assays in this study were run on an ABI 7500 FAST Real-Time PCR System (Applied Biosystems; Thermo Fisher Scientific, Reinach, Switzerland) with an initial step of 2 min at 50 °C and a denaturation for 10 min at 95 °C, followed by 45 cycles of 15 s at 95 °C and 1 min at 60 °C. All primers and probes are listed in the Table 3. In addition, DNA sequencing was performed for molecular characterization on samples randomly selected among the DNA PCR-positive samples. Selected DNA samples were used as a template in conventional PCR assays with genus- and species-specific primers for *Ehrlichia*/*Anaplasma* spp. (16S rRNA gene) [53], *A. platys* (*groEL* gene) [39], *E. canis* (*gltA* gene) [38], and *Rickettsia* spp. (*ompA*, *ompB* and *htrA* genes) [54,55,56]. Each PCR reaction consisted of 10 μL of 5× Phusion HF buffer (Finnzymes, Espoo, Finland), 400 nM each primer, 200 nM each deoxynucleotide triphosphate (dNTP) (Sigma-Aldrich, Buchs, Switzerland), 1 U Phusion DNA Polymerase (Finnzymes, Espoo, Finland), 5 μL of DNA template, and nuclease-free water (Thermo Fisher, Darmstadt, Germany) in a final volume of 50 μL. The conventional PCR assays were run on a Biometra T-Personal 48 Thermocycler (Biometra, Gottingen, Germany). All PCR reactions were performed including negative, positive and extraction controls in each run. The cycling conditions and primers for sequence analysis are listed in Table 4. Amplified PCR products were electrophoresed in 1.5% agarose gels (100 V, 45 min), pre-stained with GelRed™ DNA Stain (Biotium, Hayward, CA, USA) and visualized under UV light. The molecular weight of the obtained products was determined using the GeneRuler™ 100 bp Plus DNA Ladder (Thermo Fisher Scientific, Darmstadt, Germany) as a molecular weight marker.

### 4.4. Sequence Analysis

PCR products were purified with the QIAquick Gel Extraction Kit (Qiagen, Hilden, Germany) following the manufacturer’s instructions. The purified PCR products were cloned using a pCR 2.1 Invitrogen TOPO TA cloning kit (Thermo Fisher Scientific, Dreieich, Germany) followed by transformation into *Escherichia coli* Top 10F´ competent cells according to the manufacturer’s protocol. Plasmid DNA was extracted from the recombinant clones using a QIAprep Spin Miniprep kit (Qiagen, Hilden, Germany) and sent for sequencing in both directions with universal primers of M13 gene (M13f: 5’ – GTA AAA CGA CGG CCAG—3’; M13r: 5’ – CAG GAA ACA GCT ATG AC—3’) to a commercial laboratory (Microsynth, Balgach, Switzerland). Obtained sequences were analysed using BLAST: Basic Local Alignment Search Tool (http://blast.ncbi.nlm.nih.gov/Blast.cgi) to determine the closest similarities to corresponding sequences of the reference strains reported in the GenBank database [57]. Theoretical translation of nucleotide sequences into amino acid sequences using the ExPASy translate tool, available on the ExPASy molecular biology server (http://www.expasy.org) [58], and the protein sequences were aligned using the ClustalW, included in the package BioEdit v.7.0.0 (Ibis Biosciences, Carlsbad, CA, USA).

### 4.5. Phylogenetic Analysis 

The phylogenetic analysis was performed on the Molecular Evolutionary Genetics Analysis software package version 7.0 (MEGA7) [59], using the neighbor-joining method. Sequences were aligned using MAFFT configured for the highest accuracy and conserved regions identified [60]. After alignment, ambiguous regions (i.e., containing gaps and/or poorly aligned) were removed with Gblocks version 0.91b [61]. For the phylogenetic trees’ construction, the best-fit model of the sequence evolution was selected based on Corrected Akaike Information Criterion (cAIC) and Bayesian Information Criterion (BIC) implemented in MEGA7. For *E. canis*-*gltA* and *A. platys*-*groEL* nucleotide sequences, the Tamura 3-parameter method, as well as for *E. canis* and *A. platys 16S* rRNA nucleotide sequences the Kimura 2-parameter, showed the lowest values of cAIC and BIC, and thus were chosen for corresponding tree reconstruction. Rates’ variation across sites was fixed to “invariant and gamma distributed”. A bootstrap analysis was performed to test the stability of the trees with 1000 replicates. GenBank accession numbers for the sequences used in the analyses are given in Figure 2, Figure 3 and Figure 4.

### 4.6. Data Analysis

The obtained data were compiled and analysed with Excel 2016 software (Microsoft Corporation, WA, USA), and statistical analysis was performed using the R software (R_Development_Core_Team, 2018). The *A. platys*, *E. canis*, *Rickettsia* spp. and co-infections prevalence rates with 95% confidence intervals (CI) were calculated using a Bayesian approach based on Beta distribution, beta (s + 1; *n*-s + 1), where s = positives; *n* = tested animals. The following variables (i.e., HCT, RBC, MCV, Hb, MCH, MCHC, WBC, PLT, segmented neutrophil, lymphocyte monocyte, and eosinophil counts) were tested for statistical association with *A. platys*, *E. canis*, *Rickettsia* spp. and co-infections PCR detection using the PCR-negatives dogs as a control group. The evaluated variables were found not to be normally distributed by Shapiro–Wilks’ W test and were analysed by the non-parametric Mann–Whitney U-test. Differences were regarded significant when *p* < 0.05.

### 4.7. Ethical Approval

Ethical approval of the present study was obtained from the Ethics Committee and Animal Welfare of Centro Nacional de Sanidad Agropecuaria (CENSA), Mayabeque, Cuba. The blood and tick sampling, as well as animal handling, was carried out by registered veterinarians. For the purposes of the study, no animal was sacrificed and the field study did not involve endangered or protected species, harm or cruelty to animals.

### 4.8. Nucleotide Sequence Accession Numbers

The nucleotide sequences obtained in this study have been submitted to GenBank under accession numbers KX792089, MK506833-4 for *A. platys 16S* rRNA; MK507007-9 for *E. canis 16S* rRNA; MK509744-6 for *A. platys groEL*; MK509747-9 for *E. canis gltA* and for MK509750-1 *R. felis htrA*.

## 5. Conclusions

In conclusion, the results of this study highlight the high prevalence of vector-borne pathogens with zoonotic potential in apparently healthy shelter dogs in Cuba. The studied region predominantly comprised urban areas, which makes the zoonotic potential a particular concern for human health. The present study also represents the first report of *R. felis* in dogs from Cuba. The high canine vector-borne pathogens (CVBPs) infection prevalence observed indicates that, in the canine population of the studied region, tick-borne pathogens, such as *E. canis*, *A. platys*, *R. felis* and possibly other members of the SFGR are circulating, which are considered to be both zoonotic and pathogenic bacteria in dogs. In addition, the detection of CVBP infection was correlated with the occurrence of haematological changes and thus our findings suggest a possible long-term health impact of arthropod-borne pathogens on infected shelter dogs. The present study is important to raise a common awareness that stray dogs can serve as immediate proximal sentinels of CVBD-causing pathogens, representing a health threat that requires consideration by Cuban veterinarians and physicians. Based on our results and clinical observations, we encourage a surveillance campaign of CVBDs for monitoring and control, with special emphasis on the investigation in humans, animals and vectors, to obtain a wider epidemiological perspective focused on the One Health approach.

## Figures and Tables

**Table 1:** Real-time qPCR frequency of vector-borne pathogens detected in dogs (*n* = 100) from Cuba.

Vector-Borne Pathogen(s)	Total	%	95% IC ^a^
Total infected dogs (≥1 pathogen)	85	85.00	77.88–92.12
*Anaplasma phagocytophilum*	0	0.0	
*Anaplasma platys*	40	40.00	30.23–49.77
*Borrelia burgdorferi* s.l.	0	0.0	
*Ehrlichia canis*	62	62.00	52.32–71.68
*Rickettsia felis* **^b^**	27	27.00	18.15–35.85
Single infections	49	49.00	39.03–58.97
*Anaplasma phagocytophilum*	0	0.0	
*Anaplasma platys*	9	9.00	3.29–14.71
*Borrelia burgdorferi* s.l.	0	0.0	
*Ehrlichia canis*	29	29.00	19.95–38.05
*Rickettsia felis* **^b^**	11	11.00	4.76–17.24
Co-infections	36	36.00	26.43–45.57
*Anaplasma platys/Ehrlichia canis*	28	28.00	19.05–36.95
*Anaplasma platys/Rickettsia felis* **^b^**	11	11.00	4.76–17.24
*Ehrlichia canis/Rickettsia felis* **^b^**	13	13.00	6.29–19.71
*Anaplasma platys/Ehrlichia canis/Rickettsia felis* **^b^**	8	8.00	2.59–13.41
Non-detected	15	15.00	7.88–22.12

**a** 95% confidence interval, Yates continuity correction performed, **b** Species according to sequencing results.

**Table 2:** Results of estimated range (minimum–maximum), mean, median, standard deviation and standard error values of haematological parameters obtained from PCR-positives and non-infected shelter dogs (*n* = 90) sampled in Havana City, Cuba.

Haematological Parameters	qPCR Positive	CBC Values					*U* Test	*p* Value
	Dogs (%)	Range	Mean	Median	SD	SE		
**Haematocrit (L/L)**								
Non-infected	12 (13.33%)	0.12–0.52	0.34	0.37	0.10	0.03		
*Anaplasma platys*	8 (8.88%)	0.15–0.47	0.35	0.39	0.11	0.04	38.50	0.485
*Ehrlichia canis*	24 (26.67%)	0.13–0.53	0.34	0.36	0.11	0.02	140.5	0.920
*Rickettsia* spp.	11 (12.22%)	0.30–0.57	0.41	0.42	0.09	0.03	42	0.146
Co-infected	35 (38.89%)	0.16–0.60	0.37	0.36	0.12	0.02	191	0.651
**Haemoglobin (g/L)**								
Non-infected	12 (13.33%)	50–189	124.08	131.00	33.90	9.77		
*Anaplasma platys*	8 (8.88%)	59–172	131.30	137.50	37.70	13.79	40	0.563
*Ehrlichia canis*	24 (26.67%)	48–192	124.00	126.00	40.20	8.02	135	0.775
*Rickettsia* spp.	11 (12.22%)	111–202	148.50	150.00	28.60	8.63	39	0.103
Co-infected	35 (38.89%)	63–211	130.30	132.00	39.50	6.68	191	0.643
**RBC Count (×10^12^/L)**								
Non-infected	12 (13.33%)	1.80–7.47	5.30	5.81	1.50	0.42		
*Anaplasma platys*	8 (8.88%)	2.28–7.46	5.60	5.97	1.80	0.68	42	0.678
*Ehrlichia canis*	24 (26.67%)	1.97–7.80	5.40	5.39	1.80	0.36	140	0.906
*Rickettsia* spp.	11 (12.22%)	4.25–8.49	6.20	6.32	1.20	0.36	43	0.169
Co-infected	35 (38.89%)	2.53–9.90	5.80	5.70	1.70	0.28	190	0.493
**MCV (fL)**								
Non-infected	12 (13.33%)	51–71	65.30	65.00	5.50	1.58		
*Anaplasma platys*	8 (8.88%)	63–69	66.00	66.50	2.40	0.80	42.5	0.698
*Ehrlichia canis*	24 (26.67%)	53–89	64.80	63.50	6.90	1.45	113	0.304
*Rickettsia* spp.	11 (12.22%)	61–72	66.50	67.00	3.60	1.07	62	0.828
Co-infected	35 (38.89%)	55–74	64.40	65.00	4.80	0.81	182	0.493
**MCH (pg)**								
Non-infected	12 (13.33%)	17.1–27.6	23.80	23.50	2.60	0.76		
*Anaplasma platys*	8 (8.88%)	21.7–26.2	23.90	23.60	1.70	0.62	41	0.616
*Ehrlichia canis*	24 (26.67%)	17.3–31.7	23.40	23.10	3.20	0.67	123	0.491
*Rickettsia* spp.	11 (12.22%)	21.9–26.8	24.00	23.80	1.60	0.48	64.5	0.951
Co-infected	35 (38.89%)	17.8–27.4	22.60	22.40	2.20	0.37	126	0.042 *
**MCHC (g/L)**								
Non-infected	12 (13.33%)	332–400	363.30	361.00	15.90	4.60		
*Anaplasma platys*	8 (8.88%)	328–387	361.90	359.00	17.70	6.61	42	0.671
*Ehrlichia canis*	24 (26.67%)	320–380	354.70	357.50	15.20	3.23	109	0.247
*Rickettsia* spp.	11 (12.22%)	346–373	361.20	361.00	9.70	2.91	64	0.926
Co-infected	35 (38.89%)	324–382	350.10	347.00	16.30	2.76	119	0.027 *
**Platelets (×10^9^/L)**								
Non-infected	12 (13.33%)	60–610	307.90	303.50	205.30	59.26		
*Anaplasma platys*	8 (8.88%)	43–354	185.10	153.00	103.30	37.42	37	0.418
*Ehrlichia canis*	24 (26.67%)	35–461	138.50	102.50	104.00	20.95	73	0.018 *
*Rickettsia* spp.	11 (12.22%)	132–698	300.00	264.00	167.20	50.42	64	0.926
Co-infected	35 (38.89%)	44–480	134.40	104.00	89.90	15.20	111	0.016 *
**WBC Count (×10^9^/L)**								
Non-infected	12 (13.33%)	4.60–13.70	10.30	10.60	2.60	0.75		
*Anaplasma platys*	8 (8.88%)	4.56–18	11.60	12.50	4.40	1.35	32	0.232
*Ehrlichia canis*	24 (26.67%)	3.60–21.10	10.20	9.40	5.10	1.06	130.5	0.663
*Rickettsia* spp.	11 (12.22%)	6–19.50	11.20	11.00	4.30	1.30	63.5	0.902
Co-infected	35 (38.89%)	5.20–23.90	12.00	11.60	5.10	0.87	175	0.393
**Total Neutrophils (×10^9^/L)**								
Non-infected	12 (13.33%)	3.60–11.20	6.88	6.80	2.13	0.62		
*Anaplasma platys*	8 (8.88%)	4.30–13.50	7.58	6.35	3.70	1.33	45	0.847
*Ehrlichia canis*	24 (26.67%)	1.32–14.60	6.64	5.85	3.63	0.76	129	0.626
*Rickettsia* spp.	11 (12.22%)	3.90–14.50	7.65	6.10	3.72	1.12	64.5	0.951
Co-infected	35 (38.89%)	2.20–70	11.73	7.40	14.26	0.73	181	0.487
**Lymphocytes (×10^9^/L)**								
Non-infected	12 (13.33%)	0.70–4.20	2.74	2.74	1.06	0.31		
*Anaplasma platys*	8 (8.88%)	0.80–8.90	3.39	3.15	2.30	0.79	35	0.334
*Ehrlichia canis*	24 (26.67%)	0.50–10.80	2.42	2.25	2.05	0.43	102	0.163
*Rickettsia* spp.	11 (12.22%)	1.40–6	2.75	2.40	1.29	0.39	61.5	0.805
Co-infected	35 (38.89%)	0.80–31	4.11	2.50	5.91	0.27	186	0.566
**Monocytes (×10^9^/L)**								
Non-infected	12 (13.33%)	0.30–1.10	0.68	0.70	0.22	0.06		
*Anaplasma platys*	8 (8.88%)	0.30–1.10	0.73	0.80	0.24	0.07	33.5	0.272
*Ehrlichia canis*	24 (26.67%)	0.10–1.10	0.61	0.60	0.27	0.06	130	0.648
*Rickettsia* spp.	11 (12.22%)	0.20–1.50	0.76	0.70	0.43	0.13	65	0.975
Co-infected	35 (38.89%)	0.20–6	1.12	0.70	1.22	0.21	175	0.396
**Eosinophils (×10^9^/L)**								
Non-infected	12 (13.33%)	0.09–1.19	0.39	0.24	0.38	0.11		
*Anaplasma platys*	8 (8.88%)	0.06–1.01	0.47	0.49	0.37	0.13	41	0.616
*Ehrlichia canis*	24 (26.67%)	0.07–1.05	0.43	0.35	0.32	0.07	129	0.626
*Rickettsia* spp.	11 (12.22%)	0.09–1.57	0.53	0.32	0.51	0.15	55	0.518
Co-infected	35 (38.89%)	0.08–4	0.64	0.52	0.71	0.12	149	0.140

CBC: blood cells count; RBC: total red blood cells; MCV: mean corpuscular volume; MCHC: mean corpuscular haemoglobin concentration; MCH: mean corpuscular haemoglobin; WBC: total white blood cells; SD: standard deviation; SE: standard error; *U* test: Mann-Whitney *U* test results. * Differences statistically significant (*p* < 0.05).

**Table 3:** Primers pair and probes used in this study for the real-time TaqMan PCR (qPCR) assays.

Pathogens	Primers/Probes Sequences [5′—3′]	Target Gene	Amplicon Size	Reference
*Internal control PCR*				
cGAPDH.427p	6-FAM—CCCTCAAGATTGTCAGCAATGCCTCCT—TAMRA	cGADPH	131 bp	Sieber-Ruckstuhl et al. [48]
cGAPDH.395f	GATGGGCGTGAACCATGAG			
cGAPDH.525r	TCATGAGGCCCTCCACGAT			
*Anaplasma phagocytophilum*				
Ep.80p	6-FAM—CCTATGCATTACTCACCCGTCTGCCACT—TAMRA	*16S* rRNA	106 bp	Pusterla et al. [49]
Ep.145f	CCATTTCTAGTGGCTATCCCATACTAC			
Ep.50r	TCGAACGGATTATTCTTTATAGCTTG			
*Anaplasma platys*				
Aplat_34p	6-FAM—AGCTACGACAAAAATCCGTTCGACTTGCA—TAMRA	*16S* rRNA	75 bp	Hofmann-Lehmann et al. [50]
Aplat.14f	CTGGCGGCAAGCTTAACAC			
Aplat.89r	CGTCTGCCACTATTTATCATAGC			
*Borrelia burgdorferi* s.l.				
B.421p	6-FAM—ATGTGCATTTGGTTATATTGAGCTTGATCAGCAA—TAMRA	*flaB*	88 pb	Leutenegger et al. [51]
B.398f	GGGAAGCAGATTTGTTTGACA			
B.484r	ATAGAGCAACTTACAGACGAAATTAATAGA			
*Ehrlichia canis*				
Ec.61p	6-FAM—TCTGCCACTAACAATTTCCTATAGCCAGAGGC—TAMRA	*16S* rRNA	108 pb	Foley et al. [52]
Ec.139f	ATGGCTATTCCGTACTACTAGGTAGATTC			
Ec.32r	CATGCAAGTCGAACGGACAAT			
*Rickettsia* spp.				
CS-P	6-FAM—TGCAATAGCAAGAACCGTAGGCTGGATG—BHQ-1	*gltA*	74 pb	Stenos et al. [42]
CS-F	TCGCAAATGTTCACGGTACTTT			
CS-R	TCGTGCATTTCTTTCCATTGTG			

BHQ: black hole quencher; 6-FAM: 6-carboxyfluorescein; TAMRA: 6-carboxytetramethyl-rhodamine; c: canine.

**Table 4:** Set of primers and cycling conditions used for sequencing analysis of vector-borne pathogens detected in dogs from Cuba.

Pathogens	Primers Sequences (5′—3′)	Target Gene	Amplicon Size	Cycling Conditions *	References
*Anaplasma* spp./*Ehrlichia* spp.				40 cycles: 10 s 98 °C; 1.5 min 72 °C	
EE1	TCCTGGCTCAGAACGAACGCTGGCGGC	*16S*rRNA	1400 pb		Barlough et al. [53]
EE2	AGTCACTGACCCAACCTTAAATGGCTG				
*Anaplasma platys*				35 cycles: 10 s 98 °C; 30 s 58 °C; 1 min 72 °C	
EphplgroEL.F	ATGGTATGCAGTTTGATCGC	*groEL*	625 bp		Alberti et al. [39]
EphplgroEL.R	TCTACTCTGTCTTTGCGTTC				
*Ehrlichia canis*				35 cycles: 10 s 98 °C; 30 s 54 °C; 1 min 72 °C	
Ec.gltA.522f	CAGGAGTATATGCCTCCTGA	*gltA*	507 pb		Marsilio et al. [38]
Ec.gltA.1031r	GTTACTTTTTTCAATTGCC				
*Rickettsia* spp.				40 cycles: 10 s 98 °C; 30 s 55 °C; 1 min 72 °C	
Rr190.70p	ATGGCGAATATTTCTCCAAAA	*ompA*	532 pb		Regnery et al. [55]
Rr190.620n	AGTGCAGCATTCGCTCCCCCT				
120-M59	CCGCAGGGTTGGTAACTGC	*ompB*	862 bp	10 s 98 °C; 30 s 55 °C; 1 min 72 °C	Roux and Raoult [54]
120-807	CCTTTTAGATTACCGCCTAA				
17kD1	GCTCTTGCAACTTCTATGTT	*htrA*	434 bp	10 s 98 °C; 30 s 55 °C; 1 min 72 °C	Labruna et al. [56]
17kD2	CATTGTTCGTCAGGTTGGCG				

* all PCR reactions: 3 min 98 °C initial activation; 7 min 72 °C final extension.

## Supplementary Materials

The following are available online at https://www.mdpi.com/2076-0817/9/11/901/s1. Table S1: The correlation about tick presence and related hosts with real-time qPCR diagnostic results of vector-borne pathogens for sampled shelter dogs (n = 100) from Havana City, Cuba. Table S2: Complete blood counts (CBC) obtained for blood samples collected from shelter dogs (*n* = 90) living in Havana City, Cuba.

## References

[1] Otranto D., Dantas-Torres F., Mihalca A.D., Traub R.J., Lappin M., Baneth G. (2017). Zoonotic Parasites of Sheltered and Stray Dogs in the Era of the Global Economic and Political Crisis. Trends Parasitol..

[2] Chomel B.B. (2011). Tick-borne infections in dogs—An emerging infectious threat. Veter. Parasitol..

[3] Dumler J.S., Barbet A.F., Bekker C.P.J., Dasch G.A., Palmer G.H., Ray S.C., Rikihisa Y., Rurangirwa F.R. (2001). Reorganization of genera in the families Rickettsiaceae and Anaplasmataceae in the order Rickettsiales: Unification of some species of Ehrlichia with Anaplasma, Cowdria with Ehrlichia and Ehrlichia with Neorickettsia, descriptions of six new species combinations and designation of Ehrlichia equi and ’HGE agent’ as subjective synonyms of Ehrlichia phagocytophila. Int. J. Syst. Evol. Microbiol..

[4] Perez M., Bodor M., Zhang C., Xiong Q., Rikihisa Y. (2006). Human Infection with Ehrlichia Canis Accompanied by Clinical Signs in Venezuela. Ann. N. Y. Acad. Sci..

[5] Bouza-Mora L., Dolz G., Solórzano-Morales A., Romero-Zuñiga J.J., Salazar-Sánchez L., Labruna M.B., Aguiar D.M. (2017). Novel genotype of Ehrlichia canis detected in samples of human blood bank donors in Costa Rica. Ticks Tick Borne Dis..

[6] Little S.E. (2010). Ehrlichiosis and Anaplasmosis in Dogs and Cats. Vet. Clin. Small Anim. Pract..

[7] Lima M., Soares P., Ramos C., Araújo F., Ramos R., Souza I., Faustino M., Alves L. (2010). Molecular detection of Anaplasma platys in a naturally-infected cat in Brazil. Braz. J. Microbiol..

[8] Dahmani M., Davoust B., Benterki M.S., Fenollar F., Raoult D., Mediannikov O. (2015). Development of a new PCR-based assay to detect Anaplasmataceae and the first report of Anaplasma phagocytophilum and Anaplasma platys in cattle from Algeria. Comp. Immunol. Microbiol. Infect. Dis..

[9] Cardoso L., Tuna J., Vieira L., Yisaschar-Mekuzas Y., Baneth G. (2010). Molecular detection of Anaplasma platys and Ehrlichia canis in dogs from the North of Portugal. Veter. J..

[10] Arraga-Alvarado C.M., Parra O.C., Hegarty B.C., Breitschwerdt E.B., Qurollo B.A., Berrueta M.A. (2014). Molecular Evidence of Anaplasma platys Infection in Two Women from Venezuela. Am. J. Trop. Med. Hyg..

[11] Dantas-Torres F. (2010). Biology and ecology of the brown dog tick, Rhipicephalus sanguineus. Parasites Vectors.

[12] Margos G., Vollmer S.A., Ogden N.H., Fish D. (2011). Population genetics, taxonomy, phylogeny and evolution of Borrelia burgdorferi sensu lato. Infect. Genet. Evol..

[13] Woldehiwet Z. (2010). The natural history of Anaplasma phagocytophilum. Veter. Parasitol..

[14] Tsiodras S., Spanakis N., Spanakos G., Pervanidou D., Georgakopoulou T., Campos E., Petra T., Kanellopoulos P., Georgiadis G., Antalis E. (2017). Fatal human anaplasmosis associated with macrophage activation syndrome in Greece and the Public Health response. J. Infect. Public Health.

[15] Parola P., Paddock C.D., Socolovschi C., Labruna M.B., Mediannikov O., Kernif T., Abdad M.Y., Stenos J., Bitam I., Fournier P.-E. (2013). Update on Tick-Borne Rickettsioses around the World: A Geographic Approach. Clin. Microbiol. Rev..

[16] Vélez J.C.Q., Faccini-Martínez Á.A., González J.D.R., Díaz F.J., García R.R., Ordosgoitia P.S., Saad E.A.P., Quintero L.O., Arbeláez C.R. (2019). Fatal Rickettsia rickettsii infection in a child, Northwestern Colombia, 2017. Ticks Tick Borne Dis..

[17] Harrus S., Perlman-Avrahami A., Mumcuoglu K.Y., Morick D., Eyal O., Baneth G. (2011). Molecular detection of Ehrlichia canis, Anaplasma bovis, Anaplasma platys, Candidatus Midichloria mitochondrii and Babesia canis vogeli in ticks from Israel. Clin. Microbiol. Infect..

[18] Hamel D., Shukullari E., Rapti D., Silaghi C., Pfister K., Rehbein S. (2015). Parasites and vector-borne pathogens in client-owned dogs in Albania. Blood pathogens and seroprevalences of parasitic and other infectious agents. Parasitol. Res..

[19] Harrus S., Waner T. (2011). Diagnosis of canine monocytotropic ehrlichiosis (Ehrlichia canis): An overview. Vet. J..

[20] Ben Said M., Belkahia H., El Mabrouk N., Saidani M., Alberti A., Zobba R., Cherif A., Mahjoub T., Bouattour A., Messadi L. (2017). Anaplasma platys-like strains in ruminants from Tunisia. Infect. Genet. Evol..

[21] Navarrete M.G., Cordeiro M.D., Silva C.B., Massard C.L., López E.R., Rodríguez J.C.A., Ribeiro C.C., Fonseca-Rodríguez O., Da Fonseca A.H. (2018). Serological and molecular diagnosis of Ehrlichia canis and associated risk factors in dogs domiciled in western Cuba. Vet. Parasitol. Reg. Stud. Rep..

[22] Da Silva C.B., Santos H.A., Navarrete M.G., Ribeiro C.C.D.U., Corona B., Zaldivar M.F., Pires M.S., Peckle M., Da Costa R.L., Vitari G.L.V. (2016). Molecular detection and characterization of Anaplasma platys in dogs and ticks in Cuba. Ticks Tick Borne Dis..

[23] Maggi R.G., E Mascarelli P., Havenga L.N., Naidoo V., Breitschwerdt E.B. (2013). Co-infection with Anaplasma platys, Bartonella henselae and Candidatus Mycoplasma haematoparvum in a veterinarian. Parasites Vectors.

[24] Oteo J.A., Portillo A., Santibáñez S., Blanco J., Pérez-Martínez L., Ibarra V. (2006). Cluster of Cases of Human Rickettsia felis Infection from Southern Europe (Spain) Diagnosed by PCR. J. Clin. Microbiol..

[25] Traversa D., Di Cesare A., Simonato G., Cassini R., Merola C., Diakou A., Halos L., Beugnet F., Di Regalbono A.F. (2017). Zoonotic intestinal parasites and vector-borne pathogens in Italian shelter and kennel dogs. Comp. Immunol. Microbiol. Infect. Dis..

[26] Liu M., Ruttayaporn N., Saechan V., Jirapattharasate C., Vudriko P., Moumouni P.F.A., Cao S., Inpankaew T., Ybañez A.P., Suzuki H. (2016). Molecular survey of canine vector-borne diseases in stray dogs in Thailand. Parasitol. Int..

[27] Soares R., Ramos C.A., Pedroso T., Babo-Terra V., Cleveland H., De Araújo F. (2017). Molecular survey of Anaplasma platys and Ehrlichia canis in dogs from Campo Grande, Mato Grosso do Sul, Brazil. Anais da Academia Brasileira de Ciências.

[28] Starkey L.A., Newton K., Brunker J., Crowdis K., Edourad E.J.P., Meneus P., Little S.E. (2016). Prevalence of vector-borne pathogens in dogs from Haiti. Vet. Parasitol..

[29] Paulino P.G., Pires M.S., Da Silva C.B., Peckle M., Da Costa R.L., Vitari G.V., Vilela J.A.R., De Abreu A.P.M., Massard C.L., Santos H.A. (2018). Epidemiology of Ehrlichia canis in healthy dogs from the Southeastern region of the state of Rio de Janeiro, Brazil. Prev. Veter. Med..

[30] Inpankaew T., Hii S.F., Chimnoi W., Traub R.J. (2016). Canine vector-borne pathogens in semi-domesticated dogs residing in northern Cambodia. Parasites Vectors.

[31] Dantas-Torres F. (2008). The brown dog tick, Rhipicephalus sanguineus (Latreille, 1806) (Acari: Ixodidae): From taxonomy to control. Veter. Parasitol..

[32] Manzillo V.F., Cappiello S., Oliva G. (2006). Tick-transmitted diseases in dogs: Clinicopathological findings. Parassitologia.

[33] Bulla C., Takahira R.K., Trinca L.A., Lopes R.S., Wiedmeyer C.E. (2004). The relationship between the degree of thrombocytopenia and infection with Ehrlichia canis in an endemic area. Veter. Res..

[34] Santos F., Coppede J.D.S., Pereira A.L., Oliveira L.P., Roberto P.G., Benedetti R.B., Zucoloto L.B., Lucas F., Sobreira L., Marins M. (2009). Molecular evaluation of the incidence of Ehrlichia canis, Anaplasma platys and Babesia spp. in dogs from Ribeirão Preto, Brazil. Veter. J..

[35] De La Fuente J., Torina A., Naranjo V., Nicosia S., Alongi A., La Mantia F.P., Kocan K.M. (2006). Molecular characterization of Anaplasma platys strains from dogs in Sicily, Italy. BMC Veter. Res..

[36] Chisu V., Zobba R., Lecis R., Sotgiu F., Masala G., Foxi C., Pisu D., Alberti A. (2018). GroEL typing and phylogeny of Anaplasma species in ticks from domestic and wild vertebrates. Ticks Tick Borne Dis..

[37] Thomson K., Yaaran T., Belshaw A., Curson L., Tisi L., Maurice S., Kiddle G. (2018). A new TaqMan method for the reliable diagnosis of Ehrlichia spp. in canine whole blood. Parasites Vectors.

[38] Marsilio F., Di Martino B., Meridiani I., Bianciardi P. (2006). Direct Identification ofEhrlichia Canisby a Novel Polymerase Chain Reaction Method and Molecular Analysis of the Citrate Synthase (gltA) Gene from Various Italian Strains. J. Veter. Diagn. Investig..

[39] Alberti A., Zobba R., Chessa B., Addis M.F., Sparagano O.A.E., Parpaglia M.L.P., Cubeddu T., Pintori G., Pittau M. (2005). Equine and Canine Anaplasma phagocytophilum Strains Isolated on the Island of Sardinia (Italy) Are Phylogenetically Related to Pathogenic Strains from the United States. Appl. Environ. Microbiol..

[40] Barros-Battesti D.M., Hernandez M.R., Famadas K.M., Onofrio V.C., Beati L., Guglielmone A.A. (2009). The ixodid ticks (Acari: Ixodidae) of Cuba. Syst. Appl. Acarol..

[41] Rodríguez I., Fernández C., Sánchez L., Martinez B., Siegrist H.H., Lienhard R. (2012). Serological evidences suggest Borrelia burgdorferi sensu lato infection in Cuba. Braz. J. Infect. Dis..

[42] Stenos J., Unsworth N.B., Graves S.R. (2005). A highly sensitive and specific real-time PCR assay for the detection of spotted fever and Typhus group Rickettsiae. Am. J. Trop. Med. Hyg..

[43] Khrouf F., Sellami H., Elleuch E., Hattab Z., Ammari L., Khalfaoui M., Souissi J., Harrabi H., M’Ghirbi Y., Tiouiri H. (2016). Molecular diagnosis of Rickettsia infection in patients from Tunisia. Ticks Tick Borne Dis..

[44] Ng-Nguyen D., Hii S.-F., Hoang M.-T.T., Nguyen V.-A.T., Rees R., Stenos J., Traub R.J. (2020). Domestic dogs are mammalian reservoirs for the emerging zoonosis flea-borne spotted fever, caused by Rickettsia felis. Sci. Rep..

[45] Noda A.A., Rodriguez I., Miranda J., Mattar S., Cabezas-Cruz A. (2016). First report of spotted fever group *Rickettsia* in Cuba. Ticks Tick Borne Dis..

[46] Nicholson W.L., Allen K.E., McQuiston J.H., Breitschwerdt E.B., Little S.E. (2010). The increasing recognition of rickettsial pathogens in dogs and people. Trends Parasitol..

[47] Estrada-Peña A., Bouattour A., Camicas J., Walker A.R. (2004). Tick of Domestic Animals in Mediterranean Region: A Guide to Identification of Species.

[48] Sieber-Ruckstuhl N., Meli M., Boretti F.S., Gönczi E., Lutz H., Reusch C. (2007). Quantitative Real-time PCR for the Measurement of 11?-HSD1 and 11?-HSD2 mRNA Levels in Tissues of Healthy Dogs. Horm. Metab. Res..

[49] Pusterla N., Huder J.B., Feige K., Lutz H. (1998). Identification of a Granulocytic Ehrlichia Strain Isolated from a Horse in Switzerland and Comparison with Other Rickettsiae of the Ehrlichia phagocytophila Genogroup. J. Clin. Microbiol..

[50] Hofmann-Lehmann R., Wagmann N., Meli M.L., Riond B., Novacco M., Joekel D., Gentilini F., Marsilio F., Pennisi M.G., Lloret A. (2016). Detection of ‘Candidatus Neoehrlichia mikurensis’ and other Anaplasmataceae and Rickettsiaceae in Canidae in Switzerland and Mediterranean countries. Schweizer Archiv für Tierheilkunde.

[51] Leutenegger C.M., Pusterla N., Mislin C.N., Weber R., Lutz H. (1999). Molecular Evidence of Coinfection of Ticks with Borrelia burgdorferi Sensu Lato and the Human Granulocytic Ehrlichiosis Agent in Switzerland. J. Clin. Microbiol..

[52] Foley J., Drazenovich N., Leutenegger C.M., Chomel B.B. (2007). Association between polyarthritis and thrombocytopenia and increased prevalence of vectorborne pathogens in Californian dogs. Vet. Rec..

[53] Barlough J.E., Madigan J.E., DeRock E., Bigornia L. (1996). Nested polymerase chain reaction for detection of Ehrlichia equi genomic DNA in horses and ticks (Ixodes pacificus). Vet. Parasitol..

[54] Roux V., Raoult D. (2000). Phylogenetic analysis of members of the genus Rickettsia using the gene encoding the outer-membrane protein rOmpB (ompB). Int. J. Syst. Evol. Microbiol..

[55] Regnery R.L., Spruill C.L., Plikaytis B.D. (1991). Genotypic identification of rickettsiae and estimation of intraspecies sequence divergence for portions of two rickettsial genes. J. Bacteriol..

[56] Labruna M.B., McBride J.W., Bouyer D.H., Camargo L.M.A., Camargo E.P., Walker D.H. (2004). Molecular Evidence for a Spotted Fever GroupRickettsiaSpecies in the TickAmblyomma longirostrein Brazil. J. Med. Entomol..

[57] Johnson M., Zaretskaya I., Raytselis Y., Merezhuk Y., McGinnis S., Madden T.L. (2008). NCBI BLAST: A better web interface. Nucleic Acids Res..

[58] Artimo P., Jonnalagedda M., Arnold K., Baratin D., Csardi G., De Castro E., Duvaud S., Flegel V., Fortier A., Gasteiger E. (2012). ExPASy: SIB bioinformatics resource portal. Nucleic Acids Res..

[59] Kumar S., Stecher G., Tamura K. (2016). MEGA7: Molecular Evolutionary Genetics Analysis Version 7.0 for Bigger Datasets. Mol. Biol. Evol..

[60] Katoh K., Standley D.M. (2013). MAFFT multiple sequence alignment software version 7: Improvements in performance and usability. Mol. Biol. Evol..

[61] Castresana J. (2000). Selection of Conserved Blocks from Multiple Alignments for Their Use in Phylogenetic Analysis. Mol. Biol. Evol..

